# Environmental Exposure to Metals, Parameters of Oxidative Stress in Blood and Prostate Cancer: Results from Two Cohorts

**DOI:** 10.3390/antiox11102044

**Published:** 2022-10-18

**Authors:** Alica Pizent, Milena Anđelković, Blanka Tariba Lovaković, Tanja Živković Semren, Aleksandra Buha Djordjevic, Marija Gamulin, Vera Bonderović, Miodrag Aćimović, Zorica Bulat

**Affiliations:** 1 Analytical Toxicology and Mineral Metabolism Unit, Institute for Medical Research and Occupational Health, 10000 Zagreb, Croatia; 2 Health Center Kosovska Mitrovica, 38220 Kosovska Mitrovica, Serbia; 3 Department of Toxicology “Akademik Danilo Soldatović”, Faculty of Pharmacy, University of Belgrade, 11211 Belgrade, Serbia; 4 Department of Oncology, University Hospital Centre, 10000 Zagreb, Croatia; 5 Department of Clinical Oncology, School of Medicine, University of Zagreb, 10000 Zagreb, Croatia; 6 Clinic for Urology and Nefrology, Clinical Center of Serbia, Resavska 51, 11000 Belgrad, Serbia; 7 Faculty of Medicine, University of Belgrade, 11211 Belgrade, Serbia

**Keywords:** prostate cancer, toxic metal(loid)s, oxidative stress, antioxidant enzymes, PSA

## Abstract

We studied the potential role of exposure to various metal(oid)s (As, Cd, Cr, Hg, Ni, and Pb) in prostate cancer. Two cohorts were established: the Croatian cohort, consisting of 62 cases and 30 controls, and the Serbian cohort, consisting of 41 cases and 61 controls. Blood/serum samples were collected. Levels of investigated metal(oid)s, various parameters of oxidative stress, and prostate-specific antigen (PSA) were determined in collected samples. A comparison of the measured parameters between 103 prostate cancer patients and 91 control men from both Croatian and Serbian cohorts showed significantly higher blood Hg, SOD, and GPx levels and significantly lower serum SH levels in prostate cancer patients than in controls. Correlation analyses revealed the significant relationship between certain parameters of oxidative stress and the concentrations of the measured metal(loid)s, pointing to the possible role of metal(oid)-induced oxidative stress imbalance. Furthermore, a significant inverse relationship was found between the blood Pb and the serum PSA in prostate cancer patients, but when the model was adjusted for the impacts of remaining parameters, no significant association between the serum PSA and the measured parameters was found. The results of the overall study indicate a substantial contribution of the measured metal(loid)s to the imbalance of the oxidant/antioxidant system. Although somewhat conflicting, the results of the present study point to the possible role of investigated metal(oid)s in prostate cancer, especially for Hg, since the obtained relationship was observed for both cohorts, followed by the disturbances in oxidative stress status, which were found to be correlated with Hg levels. Nevertheless, further studies in larger cohorts are warranted to explain and confirm the obtained results.

## 1. Introduction

Prostate cancer is among the most common malignancies in men worldwide, with an estimated more than 1.4 million new cases and 375,000 deaths in 2020 [1,2]. Its incidence and mortality rates vary markedly among different populations. In Croatia, prostate cancer was the most commonly diagnosed malignancy in men, accounting for 17.6% of all newly diagnosed cancers [3], whereas in Serbia, it was the third most common cancer in men, representing 12.3% of all newly diagnosed cancers [4] in men in 2020. The estimated age-standardized mortality rate in Croatia was also higher than in Serbia in 2020, 14.7 vs. 12.0.

The risk of prostate cancer increases with age, and it primarily affects men older than 65 years of age [5,6]. It is widely accepted that multiple risk factors are linked to its development [7]. However, its etiology and pathogenesis remain poorly understood [6]. Since incidence and mortality rates vary substantially by residential geographic location across various populations, in addition to differences in race, ethnicity, screening practice, health care, and cancer registration, it has been assumed that exposure to various external risk factors may be responsible for these variations. Usually, prostate cancer progresses very slowly and can often be treated successfully, especially if diagnosed in the early stage. The diagnosis of prostate cancer is based on histopathological confirmation of prostate biopsy cores, after elevated prostate-specific antigen (PSA) or positive digital rectal examination. The PSA is mainly excreted from the normal epithelium of prostate tissue and its level rises slightly with age as the prostate gland grows bigger. However, a number of other factors can also affect PSA levels, including lifestyle and metal(loid) exposure [8,9,10,11,12,13].

Metal(loid)s are pervasive in the human environment, in soil, water and air. Non-occupational human exposure occurs mainly via contaminated food, and active and passive inhalation of tobacco smoke. Thus, they accumulate in human body for many years or even decades. Arsenic (As), lead (Pb), and mercury (Hg) are the top three chemicals that pose the most significant risk to human health according to the ATSDR 2019 Substance Priority List [14]. Furthermore, the World Health Organization (WHO) has identified As, cadmium (Cd), Pb, and Hg as four of ten chemicals of major public health concern [15]. Even at low doses, they may induce adverse health effects, particularly in the conditions of long-term exposure that are characteristic of environmental exposure. Adverse effects of environmental exposure to these metal(loid)s were observed on certain parameters of the male reproductive system [16,17,18,19]. According to the International Agency for Research on Cancer (IARC), As, Cd, chromium (Cr) (VI), nickel (Ni), and some of their compounds are classified as carcinogens to humans (group 1), while inorganic Pb and Pb compounds are classified as a probable human carcinogen (group 2A) [20]. In addition, several of these metal(loid)s are recognized as endocrine disruptor chemicals (EDCs) [16,21,22,23,24] that interfere with estrogen and androgen signaling pathways affecting the expression of genes involved in the growth and the secretory function of the prostate gland and contributing to prostate carcinogenesis [25,26]. Although the scanty epidemiologic data available indicate a possible contribution of several individual metal(loid)s to an increased risk of prostate cancer, only a few studies investigated their contribution to the increased PSA levels in presumably healthy men [10,12,13,27].

The carcinogenic effects of metal(loid)s have been related to the modification of tumor suppressor gene expression, the activation of redox-sensitive transcription factors and signaling pathways of proteins involved in cell growth, apoptosis, cell cycle regulation, DNA repair, and differentiation [28,29,30,31,32]. Although precise mechanisms of metal-induced toxicity and carcinogenicity are not well understood, oxidative stress has been recognized to have a substantial role in the activation of inflammatory mediators and other cellular processes involved in the initiation and progression of cancer [33]. The imbalance between the production of reactive oxygen species (ROS) and antioxidant defense molecules may reduce the ability of the biological system to repair oxidative damage of proteins, lipids, and DNA, contributing to the pathogenesis and the progression of age-related diseases such as prostate cancer [33,34,35]. Malignant and aging benign prostatic tissues showed an increase in oxidative DNA base lesions represented by 8-hydroxyadenine (8-oxoA) and 8-hydroxyguanine (8-oxoG) [36]. Prostate cancer patients showed significantly higher prostate tissue expression and higher urinary and leucocyte 8-hydroxyguanosine (8-OHdG) [37,38,39], and lower reduced plasma glutathione and glutathione S-transferase [39] than control subjects.

Although the importance of certain factors in the etiology of neoplasms has been recognized, their interrelationship and influence on the levels of tumor markers in subjects with prostate cancer have not been examined so far. In this study, we present data on the total concentration of As, Cd, Cr, Hg, and Pb in blood, Ni in blood, and serum of prostate cancer patients and control men in Serbia and Croatia as biomarkers of exposure. The aim of the study was to assess the role of environmental exposure to these elements in prostate cancer and to evaluate their impact on the serum PSA and the parameters of oxidative stress: total oxidative stress (TOS), total antioxidant status (TAS), the activity of superoxide dismutase (SOD) and glutathione peroxidase (GPx), advanced oxidation protein products (AOPP), and total sulfhydryl (SH) group levels in the prostate cancer patients.

## 2. Materials and Methods

### 2.1. Study Population

The study was carried out in 103 men with prostate cancer (cases) and 91 healthy men (control) who had never been occupationally exposed to metal(loid)s. The Croatian cohort consisted of 62 cases and 30 controls, and the Serbian cohort consisted of 41 cases and 61 controls. Each subject was informed about the study protocol and signed a consent form. The study was designed in accordance with the Helsinki Declaration and approved by the ethical committees of the collaborating institutions.

The Croatian study was approved by the Ethical Committee of the University Hospital Centre (no. 01/001/VG) and the Institute for Medical Research and Occupational Health (no. 01-74/2-11) in Zagreb, Croatia. Cases were men with newly diagnosed prostate cancer recruited between June 2011 and October 2013 at the Department of Oncology, the University Hospital Centre Zagreb, Croatia. The diagnosis was based on serum prostate-specific antigen (PSA) and digital rectal examination (DRP) and confirmed by histopathological evaluation. The control group consisted of healthy male volunteers randomly selected from male blood donors at the Croatian Institute for Transfusion Medicine, Zagreb, Croatia, who had no prior history of the malignant disease at the time of recruitment. For both groups, information regarding anamnestic data, sociodemographic characteristics, and lifestyle habits was collected by questionnaires and clinical records.

The Serbian study was approved by the Scientific Ethical Committee of the Institute of Urology and Nephrology and Clinical Center of Serbia (license number 526/9) and the Ethics Committee for Biomedical Research, University of Belgrade—Faculty of Pharmacy (license number 288/2). The study has carried out over a two-year period at the Institute of Urology and Nephrology, Clinical Center of Serbia in Belgrade. Patients were segregated into groups based on histopathological tissue verification by a pathologist. None of them had other malignant diseases or prostate cancer in their personal medical history. The control group was represented by healthy adult volunteers. All participants at the point of study entry responded to a standardized questionnaire administered by trained medical interviewers.

This study was not originally designed within the same project; therefore, we harmonized data by selecting the variables common to both cohorts. Although the time of sampling differs between cohorts, both cohorts had a comparable evaluation of measured metal(loid)s levels in whole blood controlled by using standard reference materials.

### 2.2. Sample Collection

Blood samples were taken from an antecubital vein and collected in a BD vacutainer for trace element testing tubes with K_2_EDTA (for whole blood samples) or without anticoagulant (for serum samples). Whole blood samples were aliquoted and stored at −20 °C until analysis. After centrifugation at 1500× *g* for 10 min, serum samples for oxidative stress parameters analysis were stored at −80 °C. Special care was taken to avoid any contamination with metals during the blood sampling, storage, and analyses.

### 2.3. Metal(loid)s Analyses

Analysis performed in Croatia. Concentrations of As, Cd, Cr, Hg, and Pb were measured in whole blood and Ni in serum samples after dilution with an aqueous solution containing 0.01 mmol/L EDTA (Sigma, St. Louis, MO, USA), 0.07 mmol/L ammonia, 0.07% (*v*/*v*) Triton X-100 (both from BDH Chemicals Ltd., Poole, UK), and 3 µg/L of internal standard [40]. The accuracy of measurements was controlled using certified reference materials (CRM), SeronormTM Trace Element Blood (Levels 1 and 2), SeronormTM Trace Elements Serum (Levels 1 and 2) (Sero AS, Billingstad, Norway), and ClinChek^®^ Whole Blood Control (Levels 1 and 2) and ClinChek^®^ Serum Control (Levels 1 and 2) (Recipe, München, Germany). Overall recoveries were 91–114% of the assigned analytical values. Measurements were made using an Agilent (Agilent Technologies, Tokyo, Japan) 7500 cx inductively coupled plasma mass spectrometer (ICPMS) with an octopole reaction system (ORS) collision/reaction cell. All measurements were performed in duplicate.

Analysis performed in Serbia. For digestion, approximately 1 mL of blood was mineralized with nitric acid and hydrogen peroxide (7 + 1) in Teflon containers according to Milestone’s recommendations (Milestone START D, SK-10T, Milestone Srl, Sorisole, Italy). After 30 min of free air digestion, Teflon containers were warmed up for 15 min to 180 °C, digested for 15 min at 180 °C, and cooled down for 30 min by microwave-assisted digestion. After digestion, samples were restored to 10 mL volume with deionized water. Cadmium and lead levels were determined by graphite furnace atomic absorption spectrophotometry (AAS GTA 120 graphite tube atomizes, 200 series AA, Agilent technologies, Santa Clara, CA, USA). Levels of arsenic, chromium, nickel, and mercury were measured using inductively coupled plasma mass spectrometry (ICPMS) 7700x (Agilent ICPMS, Santa Clara, CA, USA). The accuracy of AAS and ICP-MS analyses was validated with standard reference material (SRM) whole blood Level 2 (SeronormTM, Sero, Billingstad, Norway).

### 2.4. Oxidative Stress Parameters

The GPx (EC 1.11.1.9) activity in blood was determined using the method described by Belsten et al. [41]. The amount of glutathione oxidized by t-butyl hydroperoxide was determined by following the decrease in the β-NADPH concentration. The decrease in absorbance at 340 nm was measured by spectrophotometry. One unit of GPx is the number of micromoles of β-NADPH oxidized per minute. The results are expressed per gram of hemoglobin (U/g Hb).

The SOD (EC 1.15.1.1) activity in the erythrocytes was determined using the RanSod commercial test (Randox, Crumlin, U.K.) according to the manufacturer’s directions. This method employs xanthine and xanthine oxidase to generate superoxide radicals which react with 2-(4-iodophenyl)-3-(4-nitrophenol)-5-phenyltetrazolium chloride (I.N.T.) to form a red formazan dye. The SOD activity is then measured by the degree of inhibition of this reaction at 505 nm by spectrophotometry. One unit of SOD is that which causes a 50% inhibition of the rate of reduction of I.N.T. under the assay conditions. The results were expressed per gram of hemoglobin (U/g Hb).

Hemoglobin (Hb) in whole blood and erythrocytes was measured at 540 nm by the standard cyanmethemoglobin method using hemiglobincyanide standard (Mallinckrodt Baker B.V., Deventer, Holland). Three levels of Hb (Mallinckrodt Baker B.V., Deventer, Holland) were used to verify the analytical procedure.

The method principle for blood total oxidative status assessment (TOS) is based on the oxidizability of oxidants presented in the samples. Namely, ferric ion from *o*-dianisidine transformed to ferric ion by oxidation. Resulting color intensity (colored complex with xylenol-orange in acid medium) is directly proportional to the oxidant levels present in the sample. The results are expressed as µmol H_2_O_2_ equivalent/L.

Total antioxidant status (TAS) assessment is based on the oxidized form of 2,2′-azinobis(3-ethylbenzo-thiazoline-6-sulfonate) (ABTS) in acid medium with H_2_O_2_ (acetate buffer 30 mM, pH 3.6) [42]. Trolox, analogous to the water-soluble form of vitamin E, is used for method calibration. The degree of discoloration (colorless reduced form of ABTS to dark green ABTS^+^ ion) measured at 660 nm is proportional to the sample total antioxidant status. The results are expressed as µmol Trolox equivalent/L.

A ratio between the obtained TOS and TAS was used for the estimation of the oxidative stress degree (OSI) [43].

Superoxide dismutase (SOD, EC 1.15.1.1) inhibits the spontaneous autooxidation of epinephrine in alkaline medium (pH 10.2). This principle method described by Misra and Fridovich [44] was used for sSOD activity determination in serum samples. The results are expressed in relative units (U/L) obtained by absorbance measurement of the resulting red oxidation product at 480 nm. sSOD activity is calculated as the inhibition percentage of epinephrine auto-oxidation.

The spectrophotometry method developed by Witko-Sarsat et al. [45], quantitative estimation of advanced oxidation protein products, was used for determining AOPP. The method is a two-step absorbance measurement, first with phosphate buffer at pH 7.4 and then with acetate solution and 1.16 M KJ. The obtained difference of measured absorbance at 340 nm quantifies the AOPP value of the sample. Levels of AOPP are expressed in equivalent of chloramine T, used for calibration (µmol/L chloramine T equivalents).

Aliphatic thiol compounds in alkaline medium react with 2,2′-dinitro-5,5′-dithio-benzoic acid (DTNB) which is used for SH group assessment in serum samples [46]. The resulting yellow reaction product was measured at 412 nm whereby one mole of thiol produces one mol of *p*-nitrophenol. Redox status analyses were performed by ILAB 300 plus analyzer (Instrumentation Laboratory, Milan, Italy).

### 2.5. Statistical Analysis

Because of the skewed distribution of most of the parameters previously tested for normality with the Shapiro–Wilk test, the results are presented as median and interquartile range (IQR). Categorical data are presented as absolute or relative frequencies. Comparison between continuous variables was performed using the Mann–Whitney *U* test and categorical variables were analyzed by Chi-square (χ2) test. Spearman’s rank correlation was performed to assess a statistical association between the variables of interest. Prior to linear regression analyses, levels of PSA and metal(loid)s were logarithmically (ln) transformed. The associations between PSA and other parameters were first assessed by simple linear regression in unadjusted models. Multiple linear regression was used to investigate the combined impact of multiple metal(loid)s and other parameters on the PSA, and the following variables were introduced in the model as covariates: age (years) and BMI (kg/m^2^) as continuous variables, and smoking habits (yes/no) and alcohol consumption (yes/no) as categorical variables. Model 1 included all covariates, Model 2 included all covariates and all biomarkers of exposure to measured metal(loid)s, and Model 3 additionally included all parameters of oxidative stress. The obtained models were checked for normality of residuals (by Shapiro–Wilk test). *p* values less than 0.05 were considered statistically significant. For statistical data evaluation, we used DellTM StatisticaTM licensed statistical software package Version 13.5.0.17 (TIBCO Software Inc., Palo Alto, CA, USA) and SPSS^®^ 18.0 for Windows (SPSS Inc., Chicago, IL, USA).

## 3. Results

Demographic characteristics of prostate cancer patients and controls are presented in Table 1. Prostate cancer patients were significantly older than control men in both cohorts, and the Croatian control group was older than the Serbian control group. Therefore, a possible impact of age on the results was taken into account. Although there was no significant difference in BMI between Croatian and Serbian cohorts, cases from the Croatian cohort had a significantly lower BMI than the control, which contributed to a significant difference in BMI between the cases and the control for the overall study group (Total = Croatia + Serbia). Most participants did not smoke (78.3%) or drink (65.5%). More smokers were among control subjects and more alcohol consumers were among prostate cancer patients.

Table 2 shows concentrations of As, Cd, Cr, Hg, and Pb in the whole blood and Ni in the whole blood and serum in men with prostate cancer and the controls in the overall study group and in each cohort. When two cohorts, cases and controls, were compared, the Serbian cohort had significantly higher median levels of Cd (1.10 µg/L vs. 0.40 µg/L), Cr (1.82 µg/L vs. 1.28 µg/L), and Hg (6.47 µg/L vs. 2.49 µg/L) and lower median levels of As (0.83 µg/L vs. 1.25 µg/L) than the Croatian cohort, whereas there was no significant difference in blood Pb levels between the groups (28.40 µg/L vs. 23.35 µg/L).

When compared to the controls, prostate cancer patients had a significantly higher blood Hg level for both cohorts and for the overall study group (Table 2). In addition, prostate cancer patients from the Serbian cohort had significantly higher blood Cd and Cr and lower As, Pb, and Ni, whereas cases from the Croatian cohort had significantly higher serum Ni than controls. When we matched the study groups by age, the difference between patients and controls remained statistically significant for Hg and Ni in the Croatian cohort and for Cr, Cd, Hg, and Pb in the Serbian cohort.

Smokers had significantly higher Cd than non-smokers, whereas alcohol consumption did not contribute to the statistical difference in the investigated metals between the groups.

**Figure 1:**
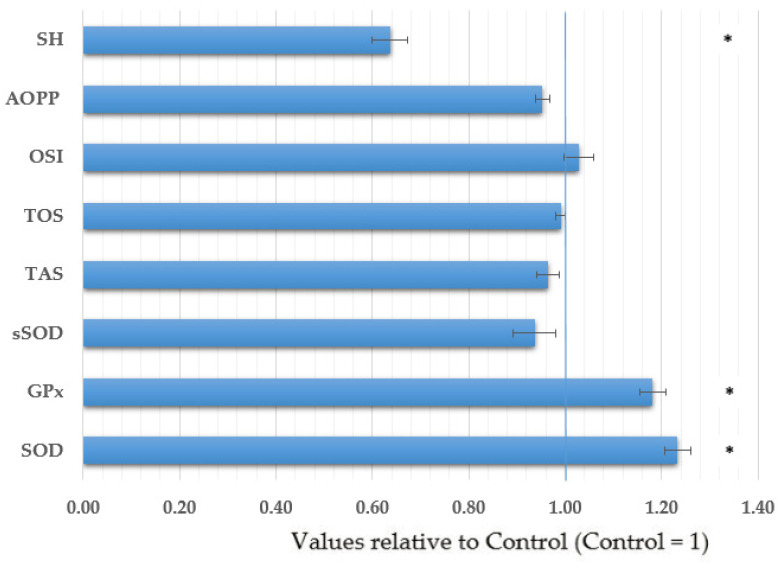
Comparison of oxidative stress parameters between men with prostate cancer and healthy men (control). The results are presented relative to control values (mean +/− SE). Relative values were calculated for each parameter separately by dividing the value of each patient by the mean value of the control group. Obtained mean and SE values are presented. * Significantly different from control (*p* < 0.05, *t*-test). Abbreviations: GPx—glutathione peroxidase activity in whole blood, SOD—superoxide dismutase activity in erythrocytes, sSOD—superoxide dismutase activity in serum, TAS—total antioxidative status, TOS—total oxidative status, OSI—oxidative stress index (the ratio of TOS to TAS), AOPP—advanced oxidation protein products levels, SH—total sulfhydryl (SH) group levels.

The case–control comparison of parameters related to oxidative stress showed a significant increase in the activity of antioxidant enzymes SOD and GPx in blood, and a significant decrease in the levels of total SH groups in the prostate cancer patients compared to control (Figure 1). These results remained statistically significant even after matching the study groups by age. There was no significant difference in other parameters of oxidative stress between the groups.

The results of Spearman’s rank correlation for the association of PSA with potentially explanatory variables (age, BMI, smoking habits and alcohol consumption, metal(loid) levels, parameters of oxidative stress) in prostate cancer patients showed a significant inverse relationship between blood Pb and PSA (R = −0.252, *p* = 0.012) when both cohorts were examined. However, when each cohort was analyzed separately, this significant association was lost. The results of Spearman’s rank correlation for the association between PSA and the parameters of oxidative stress in prostate cancer patients showed no significant association.

Table 3 shows Spearman’s correlation coefficients for the relationship between the parameters of oxidative stress and the concentrations of the measured metal(loid)s. An inverse relationship was found between serum SH and blood Cd (R = −0.206, *p* = 0.039) and Hg (R = −0.464, *p* < 0.001). A positive relationship was found between erythrocyte SOD and blood Hg (R = 0.365, *p* < 0.001) and serum Ni (R = 0.219, *p* = 0.036), between OSI and blood Hg (R = 0.223, *p* = 0.043), and between serum SH and blood Ni (0.264, *p* = 0.011). When these correlations were evaluated only among the prostate cancer patients, a positive relationship between TOS and blood Hg was found (R = 0.319, *p* = 0.048).

The results of the simple linear regression for the overall study group showed a significant inverse relationship between PSA and blood Pb (ln PSA = 3.288–0.287 ln BPb, R^2^ = 0.08, *p* = 0.005). This inverse relationship was confirmed for the Serbian cohort (ln PSA = 3.58–0.395 ln BPb, R^2^ = 0.135, *p* = 0.027), while the results in the Croatian cohort were not statistically significant (ln PSA = 2.933–1.80 ln BPb, R^2^ = 0.028, *p* = 0.169).

However, after adjusting for potentially explanatory variables (taking into account the influence of age and other metal(loid)s) in the models of multiple regression, no significant association between PSA and other measured parameters was found.

## 4. Discussion

### 4.1. Prostate Cancer and Metal(loid)s Exposure

Prostate cancer is a heterogenous disease, and lifestyle and exposure to various environmental factors are considered to contribute to the increased risk of this disease. Metal(loid)s such as As, Cd, Cr, Hg, Ni, and Pb are persistent in the environment and pervasive in the air, water, soil, and food. Hence, the general population is exposed to them daily.

Cadmium is a well-known accumulative toxicant that has a long biological half-life and low excretion levels due to the lack of efficient excretion mechanisms [47]. Apart from dietary intake, smoking is the most significant source of high-dose Cd exposure in the general population. Smokers typically absorb about 1 to 3 µg Cd/day, which is comparable to the amounts absorbed from diet [48]. Epidemiological studies found that Cd levels in prostate tissues and plasma of prostate cancer patients are significantly higher than in healthy controls [49], indicating that the accumulation of Cd in prostate tissue might be involved in the neoplastic process. To date, many experimental studies have confirmed the carcinogenic property of Cd in the human prostate in vivo and in vitro [50,51]. However, despite some suggestive findings, the majority of the epidemiological studies and meta-analysis performed so far do not provide convincing evidence that low Cd exposure may present a risk for prostate cancer development. A meta-analysis combining available data from observational studies published before October 2015 revealed that high Cd exposure is a risk factor for prostate cancer development in occupational rather than non-occupational settings. In the general population, neither urine Cd nor dietary Cd were associated with increased prostate cancer risk, and these findings were further confirmed by a subgroup analysis of studies controlling for smoking status performed to minimize possible non-Cd-mediated negative effects of tobacco smoking [52]. Similarly, a meta-analysis of 12 cohort studies and 9 case–control studies did not find any epidemiological evidence that Cd exposure may increase the risk of prostate cancer in either the general or the occupational populations [53]. Our results indicate that chronic Cd exposure, to some extent, may be related to the prostate injury. Significantly higher blood Cd in prostate cancer patients than in controls was found in the Serbian cohort, whereas no significant difference between groups was observed in the Croatian cohort. The reason for these results could not be explained.

Despite the marked progress in decreasing environmental Pb exposure, an association between blood Pb levels and cancer mortality in several populations has been indicated [54,55,56], and tobacco smoke and alcohol drinks are the main non-occupational sources of exposure. Pb accumulates over a lifetime in various organs, primarily bones, and eliminates from the body very slowly and may adversely affect various organ systems, including the male urinary tract and reproductive system, and increase the risk of cancer development. Epidemiologic studies focused on cancer risk among workers exposed to inorganic Pb found mostly only weak or no associations for lung, kidney, and brain cancers [57,58,59,60,61] but no association for prostate cancer [62,63]. Several studies indicated a positive relationship between Pb exposure and prostate cancer risk. Significantly higher concentrations of Pb in blood [64,65], serum [66], scalp hair, and nails [64] were found in prostate cancer patients than in controls. Similarly, Guzel et al. [67] found the highest levels of Pb in the blood and prostate of patients with high-grade prostatic intraepithelial neoplasia, and these levels were significantly higher than those in subjects with benign prostatic hypertrophy (BPH). Contrary to these results, our results showed a significantly lower blood Pb in prostate cancer patients compared to controls when considering the overall study group and the Serbian cohort, whereas the Croatian cases and controls had similar blood Pb levels. In agreement with our results, in multiple regression models considering the combined mixture effects of ten metal(loid)s, Lim et al. [68] did not find a significant association between serum lead and prostate cancer risk.

Drinking water naturally contaminated with As is a major source of human exposure to inorganic As, with more than 200 million people affected globally. Food, particularly rice, rice cereals and other rice products and poultry, may be additional sources of exposure to inorganic As. Inorganic As is a well-known human carcinogen and is primarily related to lung, skin, and bladder cancer [69]. A recent epidemiological study linked As exposure with prostate cancer, but with limited evidence presented in human studies [70]. Results from an in vitro study with the human prostate epithelial cell line demonstrated malignant transformation after several months of As exposure [71], but there is a lack of sufficient evidence from in vivo studies [70]. The population-based study focused on the dose–response relationship between prostate cancer and high As exposure from drinking water and recognized their interconnection in 1980 [25]. Since then, the latest studies by Ahn et al. [72] and Bulka et al. [73] confirmed a significantly higher incidence of prostate cancer in people consuming drinking water with higher As levels. Contrary to significantly higher As serum levels in prostate cancer patients demonstrated by Lim et al., the results of the present study revealed significantly lower blood As levels (*p* < 0.05) in the prostate cancer patients of the Serbian cohort in comparison to the control group. On the other hand, no significant difference between the groups was observed in the Croatian cohort. Moreover, the difference between the control group and cases lost significance if the entire study population was observed. Similar to the results obtained for Pb levels, smoking status, age dichotomy, and study design difference may contribute to the obtained results.

According to IARC, Hg(0) and inorganic Hg compounds are classified as group 3 (not classifiable according to their carcinogenicity to humans), while methylmercury compounds are in group 2B [74]. In vitro studies proposed Hg as a tumor promotor since Hg induces hypomethylation and hypermethylation of the G protein signaling, which is not a mutagenic/genotoxic metal directly affecting the gene expression [75]. In vivo study with male Wistar rats subacute orally treated with mercury chloride demonstrated increased activity of total acid phosphatase, prostatic acid phosphatase, and prostatic alkaline phosphatase as early markers of prostate cancer, in comparison to nonexposed animals [76]; however, human studies have not yet found a distinct association. In a study with prostate cancer patients, almost twofold higher Hg levels were observed in tumor tissue in comparison to healthy prostate tissues [77]. The results obtained in a National Health and Nutrition Examination Survey (NHANES) study with men aged ≥ 40 years did not recognize blood Hg as a significant predictor for PSA elevation, a tumor marker related to prostate tissue [13]. Moreover, a case–control study with prostate cancer patients did not recognize significant differences between Hg levels in toenails compared to control levels [78]. Higher blood Hg levels were observed in both Serbian and Croatian prostate cancer cohorts compared to the corresponding control groups. In accordance, higher blood Hg levels were demonstrated in prostate cancer patients in the entire study cohort in comparison to controls. As Hg naturally occurs in the environment, the general population is exposed to low Hg levels from the air, water, and food. Fish, shellfish, and marine mammals may contain higher Hg levels since methylmercury accumulates up the food chain. Another potential source is dental amalgam filling, with 0–75% of total daily Hg intake [79]. Moreover, as higher Hg levels were observed in older people [80], we can assume the possibility of an age-related increase in mercury body burden. However, metabolism alteration in a cancer cell may contribute to increased cellular uptake and/or depress the elimination of Hg.

Prior research generally confirms that Cr hexavalent form (VI) can play a crucial role in the development and progression of lung cancer in occupationally exposed workers through either inhalation or dermal absorption [81,82,83]. Concerning prostate cancer, a recent meta-analysis indicated that exposure to chromium might moderately increase the risk among exposed workers [84]. As a potent oxidizing agent, Cr (VI) compound may induce the formation of DNA–protein cross-links and lipid peroxidation, leading to oxidative stress [85]. In addition, both in vitro and in vivo evidence has shown that low doses of Cr (VI) exposure promote prostate cancer cell growth and migration by affecting the epithelial–mesenchymal transition (EMT) pathway, which can lead to tumor progression and metastasis [86]. In the Serbian cohort, we observed significantly higher Cr levels in patients diagnosed with prostate cancer compared to the control group, but at the same time, we did not find statistically significant results when the entire study population was observed. Similar to findings from the Serbian cohort, in a study by Zhang et al., Cr was detected in higher concentrations in the blood of prostate cancer patients, associated with the progression of the disease [86]. However, another study that performed trace metal analysis in Nigerian patients with prostate cancer, BPH, and healthy controls showed no significant relationship between Cr levels and the risk of developing prostate cancer [87].

Human and animal data show that exposure to both soluble and insoluble Ni compounds can lead to lung and nasal cancers [20]. On the contrary, few studies have investigated the role of Ni in the development of prostate cancer. One of the limitations is the Ni compound’s poor detectability, limiting the ability to evaluate the association between this metal and prostate cancer risk [68]. The results of a case–control study by Blanc-Lapierre et al. [88] indicate that prolonged exposure to Ni, or Ni and Cr, might increase the risk for an aggressive form of prostate cancer. In the Croatian cohort, blood serum Ni concentrations of the prostate cancer group were significantly higher than in control subjects. On the contrary, a notably lower whole blood Ni level was demonstrated in the prostate cancer group compared to the healthy controls in the Serbian cohort. As we previously mentioned, not enough studies have examined the relationship between levels of Ni in the human body and their association with prostate cancer risk. The relationship between Ni, selenium, and calcium and prostate cancer was studied by Çelen et al. [89]. They found a significantly lower Ni level in cancerous tissues than in benign prostate hyperplasia tissues. Chang et al. [90] observed a higher level of Ni (*p* < 0.001) in the blood of patients with benign prostate hyperplasia compared to controls. On the contrary, other studies reported notably higher Ni levels in malign prostate tissues [91,92,93].

### 4.2. Prostate Cancer and Oxidative Stress Parameters

Oxidative stress related to aging and inflammatory processes is considered to be one of the mechanisms that triggers the chain of reactions involved in the development and progression of prostate cancer [33,34].

Lower expression of Cu, Zn-SOD, Mn-SOD, and catalase in intraepithelial neoplasia and prostate cancer than in benign epithelium implicated the role of oxidative stress in an early event in prostate carcinogenesis [94]. In addition, increased lipid peroxidation with concomitant decreased [95,96] or increased [97] activities of antioxidant enzymes contribute to the evidence that an altered prooxidant–antioxidant balance is a critical issue in the increased risk of prostate cancer development, particularly during the early stages [39,98]. It has been found that prostate cancer patients had significantly lower plasma GSH and GPx than control subjects, whereas erythrocyte GPx activity was similar in both groups [99]. Prostate cancer patients enrolled in our studies had significantly higher erythrocyte Cu, Zn-SOD, and whole blood GPx activity and lower levels of total serum SH than control subjects. These results suggest the activation of the antioxidant defense system as a mechanism of protecting the cells against ROS. In particular, lower SH levels obtained in the present study undoubtedly confirmed the increased production of free radicals and the consequent increased consumption of antioxidants to balance the disturbed homeostasis of the oxidative status. Similar results were obtained in other studies. Significantly higher erythrocyte SOD was measured in prostate [97] and testicular [100] cancer patients compared to the control. Additionally, the depletion of GSH in testicular cancer patients was observed [100]. In the current study, decreased SH levels were associated with increased blood Cd and Hg levels. However, serum total SH levels in a large population-based Norvegian Tromsø study were not statistically significantly associated with prostate cancer risk [101]. GSH is one of the most abundant non-protein thiols (SH) in the human body and is able to react with free radicals and trap redox-active metal ions. Other sources of thiols are albumin and thioredoxin, which contribute to the total thiol levels in serum. These thiols play a critical role in maintaining the prooxidant–antioxidant balance in the cells.

Long-term exposure to various toxic metals may disturb the prooxidant–antioxidant balance due to increased generation of free radicals, inactivation of antioxidant enzymes, or excessive consumption of antioxidants [102]. In the present study, blood Hg was positively correlated with erythrocyte SOD and OSI, while serum Ni was positively correlated with erythrocyte SOD. When these correlations were evaluated only among the prostate cancer patients, a positive relationship between blood Hg and serum TOS was found.

Cd and Pb are redox-inactive metals that may induce oxidative stress indirectly. They can interfere with the activity and structural function of several enzymes and proteins, displacing essential elements from their active site. Cd overburden may lead to the depletion of antioxidant reserves in cells, with a decrease in intracellular levels of antioxidant enzymes SOD and GPx, and activate redox-sensitive transcription factors such as NF-κB [103,104]. Other molecular mechanisms that may favor carcinogenesis include deregulation of cell proliferation, disturbance of tumor suppression functions and DNA repair processes, as well as alterations of DNA methylation [105,106]. Studies investigating low (nanomolar) Cd concentrations suggest that Cd may act as a hormone disruptive agent and activator of signal transduction pathways that promote cell growth [107,108,109]. Significantly higher blood Pb levels in prostate cancer patients compared to controls were significantly correlated with increased thiobarbituric acid-reactive substances and decreased glutathione [65]. The levels of tissue GSH in malign prostate cancer were significantly lower than in BPH patients, whereas MDA in plasma and prostate tissue in patients with different grades of prostate carcinoma were significantly higher than in BPH subjects [67]. The authors also found significant positive correlations between blood and tissue Pb levels with MDA in plasma and tissue. Lower serum concentrations of essential elements manganese, iron, and zinc measured in cancer patients were suggested to contribute to the disturbance of antioxidant defense and increased susceptibility to cancer development [66]. Oxidative stress is suggested as one of the crucial mechanisms in Hg-induced pathologies [31,110]. Hg has a strong ability to deplete intracellular thiols (especially glutathione) and bind to thiol groups on proteins. The precise mechanism for ROS production by Hg is unknown but depends on Hg’s physical and chemical form. Mercury in the blood usually indicates exposure to organic methylmercury associated with eating fish or elemental mercury vapor. The high affinity of the methylmercury for selenohydryl groups, thiols, and selenides leads to the impairment of antioxidant enzymes and proteins’ structure and function [111]. On the other hand, methylmercury in nonlethal doses can activate an antioxidant transcription factor Nrf2-dependent antioxidant response [112].

In vitro, Ni compounds have been shown to cause indirect DNA damage by increasing the formation of ROS or the inhibition of DNA repair enzymes and influence gene expression by epigenetic mechanisms such as DNA methylation [113]. Further studies need to elucidate these mechanisms.

### 4.3. Impact of Metal(loid)s and Oxidative Stress on the PSA

PSA has been used as a routine biological tumor marker for the early detection of prostate cancer because men with higher PSA have an increased chance of having prostate cancer. However, PSA alone does not appear to have sufficient specificity for clinically important cancers. Moreover, other factors such as lifestyle and metal(loid) exposure could also affect PSA levels [8,9,10,11,12,13]. Therefore, we explored the possible relationship between several metal(loid)s and parameters of oxidative stress on the levels of serum PSA in prostate cancer patients from Croatia and Serbia. An inverse relationship between blood Pb and PSA obtained by the results of Spearman’s rank correlation and simple linear regression in unadjusted models (the results obtained for the overall study group and for the Serbian cohort) was not confirmed in the multiple regression after adjusting for the impact of the remaining variables.

In our previous study in apparently healthy men [10], the combined impact of age, smoking, alcohol consumption, blood Cd and Pb, and serum copper, selenium, and zinc on serum PSA was investigated. Our results obtained in relatively young men (aged 21 years to 40 years) with low-level environmental Pb exposure showed a significant positive relationship between blood Pb and PSA. A dose–response relationship between Cd body burden and serum PSA was found among prostate cancer cases with abnormal PSA [114]. Although presumably healthy U.S. men with elevated serum PSA had significantly higher blood Cd and Pb levels, no association was found after adjusting for potential confounders [13]. Additionally, no association was observed between urinary Cd levels and elevated PSA among presumably healthy men [115]. The authors suggested that the lack of association between Cd and/or Pb levels and elevated PSA could be due to low exposures to these metals in the studied population, through either diet or inhalation, in comparison to populations where exposure to these toxic elements may be prevalent [13]. In a study by van Wijngaarden et al. [115], the effect of Cd on PSA levels was only observed when dietary Zn intake levels were low (<12.7 mg/day), indicating the potential impact of Zn on Cd-induced prostatic cellular injury. No association between blood Hg and urinary As levels and higher levels of PSA was observed in healthy U.S. men [13].

## 5. Conclusions

In the current study, the comparison of measured parameters between prostate cancer patients and control showed an association between blood metal(loid) concentration and the parameters of oxidative stress and antioxidant defense. The results obtained were somewhat conflicting, varying in metal(oid)s and by the cohort group studied. This can be explained by the variety in the study groups as well as metal(oid) imbalance that can be amplified by the prostate cancer condition itself. The possible reasons for these results may also be the difference in natural sources and levels of environmental/industrial exposure to the investigated metal(loid)s in Croatia and Serbia. For example, both coal and copper ore in Serbia have substantial naturally occurring contents of Cd and Hg [116], and coal mining and mineral processing together with fumes from coal power plants may contribute to Cd and Hg exposure of the Serbian cohort. In Croatia, the spatial distribution of metal(loid)s in the surface part of the soil showed enlarged concentrations of naturally occurring As, Cd, and Ni in some Croatian regions [117], contributing to daily dietary intake of these metal(loid)s. However, the results obtained for the levels of Hg were consistent in both cohorts, pointing to significantly higher levels of Hg in cases when compared to levels in controls, even after matching the groups based on age. The presumed role of Hg in prostate cancer can be partly explained by the disturbances in oxidative stress status, which were found to be correlated with Hg levels. However, this association was not found for the levels of Hg and PSA. Further studies in larger cohorts are needed to explain and confirm the obtained results.

## Figures and Tables

**Table 1:** Demographic characteristics of prostate cancer patients and controls.

Variable	Total		Croatian Cohort		Serbian Cohort	
	Cases (n = 103)	Control (n = 91)	Cases (n = 62)	Control (n = 30)	Cases (n = 41)	Control (n = 61)
Age, years	68 (44–81)	45 (24–77)	70 (54–81)	54 (45–66)	67 (44–78)	38 (24–77)
BMI, kg/m^2^	26.7 (19.1–35.1)	28.7 (20.1–40.1)	26.5 (19.5–35.1)	28.8 (23.8–37.4)	26.8 (22.0–34.6)	28.7 (20.1–40.1)
Active smoking status, N (%)	17 (16.5)	25 (27.5)	9 (14.3)	8 (25.8)	8 (19)	17 (27.4)
Current alcohol use, N (%)	40 (38.8)	27 (29.7)	18 (28.6)	8 (25.8)	22 (52.4)	19 (30.6)
PSA ^a^, ng/mL	11.99 (0.76–134.00)		10.60 (2.34–134.00)		13.81 (0.76–108.66)	

Data are presented as median (range) or total number (percent). ^a^ PSA values for participants in control groups are not measured and considered to be lower than the reference values < 4.

**Table 2:** Concentration of arsenic (As), cadmium (Cd), chromium (Cr), mercury (Hg), nickel (Ni), and lead (Pb) in whole blood and Ni in serum of men with prostate cancer (cases) and controls.

	Total		Croatian Cohort		Serbian Cohort	
	Cases (n = 103)	Control (n = 91)	Cases (n = 62)	Control (n = 30)	Cases (n = 41)	Control (n = 61)
Metal(loid) (µg/L)						
***Blood arsenic***						
median	0.93	1.09	1.17	1.44	0.50	1.01
IQR	0.46–2.31	0.71–1.97	0.72–2.93	0.86–2.47	0.29–1.09	0.43–1.62
	*p* = 0.35		*p* = 0.44		***p* = 0.04**	
***Blood cadmium***						
median	0.80	0.80	0.41	0.40	1.30	1.00
IQR	0.32–1.36	0.49–1.30	0.27–0.77	0.24–0.83	1.10–1.90	0.80–1.40
	*p* = 0.66		*p* = 0.88		***p* = 0.0003**	
***Blood chromium***						
median	1.41	1.27	1.28	1.28	2.56	1.19
IQR	1.11–1.93	1.05–1.80	1.04–1.45	1.23–1.47	1.57–4.11	0.90–3.30
	*p* = 0.17		*p* = 0.22		***p* = 0.005**	
***Blood lead***						
median	20.75	32.00	23.35	22.34	15.70	37.70
IQR	11.77–37.56	21.2–47.24	14.95–44.13	15.60–31.94	7.90–21.65	28.00–53.90
	***p* = 0.0001**		*p* = 0.46		***p* < 0.0001**	
***Blood mercury***						
median	7.10	2.92	3.49	1.15	13.51	3.72
IQR	2.58–14.33	1.08–4.74	1.75–8.10	0.48–2.40	7.66–20.76	2.16–5.82
	***p <* 0.0001**		***p* < 0.0001**		***p* < 0.0001**	
***Blood nickel***						
median					10.56	17.57
IQR					5.87–16.44	6.84–39.73
					***p* = 0.03**	
***Serum nickel***						
median			0.90	0.69		
IQR			0.65–1.10	0.63–0.75		
			***p* = 0.0006**			

Significance of the difference between the groups (*p* < 0.05, Mann–Whitney U test). Results are presented as median (interquartile range, IQR). Significant values (*p* < 0.05) are presented in bold font.

**Table 3:** Spearman’s correlation coefficients for the relationship between the parameters of oxidative stress and the concentrations of the measured metal(loid)s in men with prostate cancer and controls.

Parameter	BAs	BCd	BCr	BPb	BHg	BNi	SNi
	(µg/L)						
SOD (U/g Hb)	−0.040	0.113	−0.126	−0.067	**0.365**	-	**0.219**
GPx (U/g Hb)	−0.099	0.012	−0.149	0.050	0.102	-	0.125
TAS (µmol/L)	0.135	0.085	−0.002	0.060	−0.214	−0.110	-
TOS (µmol/L)	0.077	0.057	−0.062	0.049	0.016	0.006	-
OSI	−0.113	−0.073	−0.035	−0.034	**0.223**	0.090	-
AOPP (µmol/L chloramine T equivalents)	−0.089	−0.145	−0.003	0.066	−0.091	−0.090	-
SH (mmol/L)	0.177	**−0.206**	−0.099	0.184	**−0.464**	**0.264**	-
sSOD (U/L)	0.081	0.034	−0.103	0.137	−0.055	0.063	-

Significant values (*p* < 0.05) are presented in bold font.

## Data Availability

The data presented in this study are available on request from the corresponding author. The availability of the data is restricted to investigators based in academic institutions.

## References

[1] IARC IARC 2021. https://gco.iarc.fr/today/data/factsheets/cancers/27-prostate-fact-sheet.pdf.

[2] Sung H., Ferlay J., Siegel R.L., Laversanne M., Soerjomataram I., Jemal A., Bray F. (2021). Global cancer statistics 2020: Globocan estimates of incidence and mortality worldwide for 36 cancers in 185 countries. CA Cancer J. Clin..

[3] International Agency for Research on Cancer Croatia Fact Sheet Source: Globocan 2020. https://gco.iarc.fr/today/data/factsheets/populations/191-croatia-fact-sheets.pdf.

[4] International Agency for Research on Cancer Serbia Fact Sheet Source: Globocan 2020. https://gco.iarc.fr/today/data/factsheets/populations/688-serbia-fact-sheets.pdf.

[5] Perdana N.R., Mochtar C.A., Umbas R., Hamid A.R.A. (2016). The risk factors of prostate cancer and its prevention: A literature review. Acta Med. Indones..

[6] Rawla P. (2019). Epidemiology of Prostate Cancer. World J. Oncol..

[7] Giovannucci E., Liu Y., Platz E.A., Stampfer M.J., Willett W.C. (2007). Risk factors for prostate cancer incidence and progression in the health professionals follow-up study. Int. J. Cancer.

[8] Karunasinghe N., Minas T.Z., Bao B.-Y., Lee A., Wang A., Zhu S., Masters J., Goudie M., Huang S.-P., Jenkins F.J. (2022). Assessment of factors associated with psa level in prostate cancer cases and controls from three geographical regions. Sci. Rep..

[9] Macke A.J., Petrosyan A. (2022). Alcohol and prostate cancer: Time to draw conclusions. Biomolecules.

[10] Pizent A., Čolak B., Kljaković Gašpić Z., Telišman S. (2009). Prostate-specific antigen (PSA) in serum in relation to blood lead concentration and alcohol consumption in men. Arch. Ind. Hyg. Toxicol..

[11] Tarantino G., Crocetto F., Vito C.D., Martino R., Pandolfo S.D., Creta M., Aveta A., Buonerba C., Imbimbo C. (2021). Clinical factors affecting prostate-specific antigen levels in prostate cancer patients undergoing radical prostatectomy: A retrospective study. Future Sci. OA.

[12] Wu C.-C., Pu Y.S., Wu H.-C., Yang C.-Y., Chen Y.-C. (2011). Reversed association between levels of prostate specific antigen and levels of blood cadmium and urinary cadmium. Chemosphere.

[13] Wu H., Wang M., Raman J.D., McDonald A.C. (2021). Association between urinary arsenic, blood cadmium, blood lead, and blood mercury levels and serum prostate-specific antigen in a population-based cohort of men in the united states. PLoS ONE.

[14] Agency for Toxic Substance and Disease Registry (ATSDR) Case Studies in Environmental Medicine (CSEM): Lead Toxicity. https://www.atsdr.cdc.gov/csem/lead/docs/CSEM-Lead_toxicity_508.pdf.

[15] World Health Organization Action Is Needed on Chemicals of Major Public Health Concern 2010. http://www.who.int/ipcs/features/10chemicals_en.pdf.

[16] Baralić K., Javorac D., Marić Đ., Đukić-Ćosić D., Bulat Z., Antonijević Miljaković E., Anđelković M., Antonijević B., Aschner M., Buha Djordjevic A. (2022). Benchmark dose approach in investigating the relationship between blood metal levels and reproductive hormones: Data set from human study. Environ. Int..

[17] Pizent A., Tariba B., Živković T. (2012). Reproductive toxicity of metals in men. Arch. Ind. Hyg. Toxicol..

[18] Ren J., Cui J., Chen Q., Zhou N., Zhou Z., Zhang G., Wu W., Yang H., Cao J. (2020). Low-level lead exposure is associated with aberrant sperm quality and reproductive hormone levels in chinese male individuals: Results from the marhcs study low-level lead exposure is associated with aberrant sperm quality. Chemosphere.

[19] Tariba Lovaković B. (2020). Cadmium, Arsenic, and Lead: Elements Affecting Male Reproductive Health. Curr. Opin. Toxicol..

[20] NTP National Toxicology Program (2016). Report on Carcinogens.

[21] Buha-Đorđević A., Anđelković M., Kačavenda E., Javorac D., Antonijević-Miljaković E., Marić Đ., Baralić K., Đukić-Ćosić D., Ćurčić M., Antonijević B. (2021). Cadmium levels in human breast tissue and estradiol serum levels: Is there a connection?. Arhiv Farm..

[22] Dyer C.A., Gore A.C. (2007). Heavy Metals as Endocrine-Disrupting Chemicals. Endocrine-Disrupting Chemicals.

[23] Iavicoli I., Fontana L., Bergamaschi A. (2009). The effects of metals as endocrine disruptors. J. Toxicol. Environ. Health Part B.

[24] Pizent A. (2021). Developmental toxicity of endocrine-disrupting chemicals: Challenges and future directions. Arhiv Farm..

[25] Benbrahim-Tallaa L., Webber M.M., Waalkes M.P. (2007). Mechanisms of acquired androgen independence during arsenic-induced malignant transformation of human prostate epithelial cells. Environ. Health Perspect..

[26] Vella V., Malaguarnera R., Lappano R., Maggiolini M., Belfiore A. (2017). Recent views of heavy metals as possible risk factors and potential preventive and therapeutic agents in prostate cancer. Mol. Cell. Endocrinol..

[27] Andreucci A., Mocevic E., Jönsson B.A., Giwercman A., Giwercman Y.L., Toft G., Lundh T., Bizzaro D., Specht I.O., Bonde J.P. (2015). Cadmium may impair prostate function as measured by prostate specific antigen in semen: A cross-sectional study among european and inuit men. Reprod. Toxicol..

[28] Buha A., Wallace D., Matovic V., Schweitzer A., Oluic B., Micic D., Djordjevic V. (2017). Cadmium exposure as a putative risk factor for the development of pancreatic cancer: Three different lines of evidence. BioMed Res. Int..

[29] Mortoglou M., Manić L., Buha Djordjevic A., Bulat Z., Đorđević V., Manis K., Valle E., York L., Wallace D., Uysal-Onganer P. (2022). Nickel’s role in pancreatic ductal adenocarcinoma: Potential involvement of MicroRNAs. Toxics.

[30] Mortoglou M., Wallace D., Buha Djordjevic A., Djordjevic V., Arisan E.D., Uysal-Onganer P. (2021). MicroRNA-regulated signaling pathways: Potential biomarkers for pancreatic ductal adenocarcinoma. Stresses.

[31] Valko M., Morris H., Cronin M.T.D. (2005). Metals, toxicity and oxidative stress. Curr. Med. Chem..

[32] Wallace D.R., Taalab Y.M., Heinze S., Tariba Lovaković B., Pizent A., Renieri E., Tsatsakis A., Farooqi A.A., Javorac D., Andjelkovic M. (2020). Toxic-metal-induced alteration in mirna expression profile as a proposed mechanism for disease development. Cells.

[33] Han C., Wang Z., Xu Y., Chen S., Han Y., Li L., Wang M., Jin X., Stockinger H. (2020). Roles of reactive oxygen species in biological behaviors of prostate cancer. BioMed Res. Int..

[34] Minelli A., Bellezza I., Conte C., Culig Z. (2009). Oxidative stress-related aging: A role for prostate cancer?. Biochim. Biophys. Acta (BBA)—Rev. Cancer.

[35] Schieber M., Chandel N.S. (2014). ROS function in redox signaling and oxidative stress. Curr. Biol..

[36] Malins D.C., Johnson P.M., Barker E.A., Polissar N.L., Wheeler T.M., Anderson K.M. (2003). Cancer-related changes in prostate DNA as men age and early identification of metastasis in primary prostate tumors. Proc. Natl. Acad. Sci. USA..

[37] Miyake H., Hara I., Kamidono S., Eto H. (2004). Oxidative DNA damage in patients with prostate cancer and its response to treatment. J. Urol..

[38] Ohtake S., Kawahara T., Ishiguro Y., Takeshima T., Kuroda S., Izumi K., Miyamoto H., Uemura H. (2018). Oxidative Stress Marker 8-Hydroxyguanosine is more highly expressed in prostate cancer than in benign prostatic hyperplasia. Mol. Clin. Oncol..

[39] Shukla S., Srivastava J.K., Shankar E., Kanwal R., Nawab A., Sharma H., Bhaskaran N., Ponsky L.E., Fu P., MacLennan G.T. (2020). Oxidative stress and antioxidant status in high-risk prostate cancer subjects. Diagnostics.

[40] Živković T., Tariba B., Pizent A. (2014). Multielement analysis of human seminal plasma by octopole reaction cell ICP-MS. J. Anal. At. Spectrom..

[41] Belsten J.L., Wright A.J. (1995). European community—FLAIR common assay for whole-blood glutathione peroxidase (GSH-Px); Results of an inter-laboratory trial. Eur. J. Clin. Nutr..

[42] Erel O. (2004). A novel automated direct measurement method for total antioxidant capacity using a new generation, more stable ABTS radical cation. Clin. Biochem..

[43] Aycicek A., Erel O. (2007). Total oxidant/antioxidant status in jaundiced newborns before and after phototherapy. J. Pediatr..

[44] Misra H.P., Fridovich I. (1972). The role of superoxide anion in the autoxidation of epinephrine and a simple assay for superoxide dismutase. J. Biol. Chem..

[45] Witko-Sarsat V., Friedlander M., Capeillère-Blandin C., Nguyen-Khoa T., Nguyen A.T., Zingraff J., Jungers P., Descamps-Latscha B. (1996). Advanced oxidation protein products as a novel marker of oxidative stress in uremia. Kidney Int..

[46] Ellman G.L. (1959). Tissue Sulfhydryl Groups. Arch. Biochem. Biophys..

[47] Cui Z.-G., Ahmed K., Zaidi S.F., Muhammad J.S. (2021). Ins and outs of cadmium-induced carcinogenesis: Mechanism and prevention. Cancer Treat. Res. Commun..

[48] Järup L., Åkesson A. (2009). Current status of cadmium as an environmental health problem. Toxicol. Appl. Pharmacol..

[49] Zhang L., Zhu Y., Hao R., Shao M., Luo Y. (2016). Cadmium levels in tissue and plasma as a risk factor for prostate carcinoma: A meta-analysis. Biol. Trace Elem. Res..

[50] Waalkes M.P. (2000). Cadmium carcinogenesis in review. J. Inorg. Biochem..

[51] Zimta A.-A., Schitcu V., Gurzau E., Stavaru C., Manda G., Szedlacsek S., Berindan-Neagoe I. (2019). Biological and molecular modifications induced by cadmium and arsenic during breast and prostate cancer development. Environ. Res..

[52] Ju-Kun S., Yuan D.-B., Rao H.-F., Chen T.-F., Luan B.-S., Xu X.-M., Jiang F.-N., Zhong W.-D., Zhu J.-G. (2016). Association between cd exposure and risk of prostate cancer: A prisma-compliant systematic review and meta-analysis. Medicine.

[53] Chen C., Xun P., Nishijo M., Carter S., He K. (2016). Cadmium exposure and risk of prostate cancer: A meta-analysis of cohort and case-control studies among the general and occupational populations. Sci. Rep..

[54] Kim M.-G., Ryoo J.-H., Chang S.-J., Kim C.-B., Park J.-K., Koh S.-B., Ahn Y.-S. (2015). Blood lead levels and cause-specific mortality of inorganic lead-exposed workers in South Korea. PLoS ONE.

[55] Li S., Wang J., Zhang B., Liu Y., Lu T., Shi Y., Shan G., Dong L. (2018). Urinary lead concentration is an independent predictor of cancer mortality in the U.S. general population. Front. Oncol..

[56] Schober S.E., Mirel L.B., Graubard B.I., Brody D.J., Flegal K.M. (2006). Blood lead levels and death from all causes, cardiovascular disease, and cancer: Results from the NHANES III mortality study. Environ. Health Perspect..

[57] Ahn J., Park M.Y., Kang M.-Y., Shin I.-S., An S., Kim H.-R. (2020). Occupational lead exposure and brain tumors: Systematic review and meta-analysis. Int. J. Environ. Res. Public. Health.

[58] Fu H., Boffetta P. (1995). Cancer and occupational exposure to inorganic lead compounds: A meta-analysis of published data. Occup. Environ. Med..

[59] Liao L.M., Friesen M.C., Xiang Y.-B., Cai H., Koh D.-H., Ji B.-T., Yang G., Li H.-L., Locke S.J., Rothman N. (2016). Occupational lead exposure and associations with selected cancers: The Shanghai men’s and women’s health study cohorts. Environ. Health Perspect..

[60] Steenland K., Boffetta P. (2000). Lead and cancer in humans: Where are we now?. Am. J. Ind. Med..

[61] Wynant W., Siemiatycki J., Parent M.-É., Rousseau M.-C. (2013). Occupational exposure to lead and lung cancer: Results from two case-control studies in Montreal, Canada. Occup. Environ. Med..

[62] Doolan G., Benke G., Giles G. (2014). An update on occupation and prostate cancer. Asian Pac. J. Cancer Prev..

[63] Fritschi L., Glass D.C., Tabrizi J.S., Leavy J.E., Ambrosini G.L. (2007). Occupational risk factors for prostate cancer and benign prostatic hyperplasia: A case-control study in Western Australia. Occup. Environ. Med..

[64] Qayyum M.A., Shah M.H. (2014). Comparative study of trace elements in blood, scalp hair and nails of prostate cancer patients in relation to healthy donors. Biol. Trace Elem. Res..

[65] Siddiqui M.K.J., Srivastava S., Mehrotra P.K. (2002). Environmental exposure to lead as a risk for prostate cancer. Biomed. Environ. Sci..

[66] Kaba M., Pirincci N., Yuksel M.B., Gecit I., Gunes M., Ozveren H., Eren H., Demir H. (2014). Serum levels of trace elements in patients with prostate cancer. Asian Pac. J. Cancer Prev..

[67] Guzel S., Kiziler L., Aydemir B., Alici B., Ataus S., Aksu A., Durak H. (2012). Association of Pb, Cd, and Se concentrations and oxidative damage-related markers in different grades of prostate carcinoma. Biol. Trace Elem. Res..

[68] Lim J.T., Tan Y.Q., Valeri L., Lee J., Geok P.P., Chia S.E., Ong C.N., Seow W.J. (2019). Association between serum heavy metals and prostate cancer risk—A multiple metal analysis. Environ. Int..

[69] IARC Working Group on the Evaluation of Carcinogenic Risks to Humans (2012). Arsenic, Metals, Fibres and Dusts.

[70] IARC Monographs on the Evaluation of Carcinogenic Risks to Humans (2012). A Review of Human Carcinogens. Part C: Arsenic, Metals, Fibres, and Dusts. Vol 100C.

[71] Benbrahim-Tallaa L., Waalkes M.P. (2008). Inorganic arsenic and human prostate cancer. Environ. Health Perspect..

[72] Ahn J., Boroje I.J., Ferdosi H., Kramer Z.J., Lamm S.H. (2020). Prostate cancer incidence in U.S. counties and low levels of arsenic in drinking water. Int. J. Environ. Res. Public Health.

[73] Bulka C.M., Jones R.M., Turyk M.E., Stayner L.T., Argos M. (2016). Arsenic in drinking water and prostate cancer in Illinois Counties: An ecologic study. Environ. Res..

[74] IARC (2022). Agents Classified by the IARC Monographs, Volumes 1–130—IARC Monographs on the Identification of Carcinogenic Hazards to Humans. https://monographs.iarc.who.int/Agents-Classified-by-the-Iarc/.

[75] Zefferino R., Piccoli C., Ricciardi N., Scrima R., Capitanio N. (2017). Possible mechanisms of mercury toxicity and cancer promotion: Involvement of gap junction intercellular communications and inflammatory cytokines. Oxid. Med. Cell. Longev..

[76] Akintunde J.K., Babaita A.K. (2017). Effect of PUFAs from Pteleopsis suberosa stem bark on androgenic enzymes, cellular ATP and prostatic acid phosphatase in mercury chloride—Exposed rat. Middle East Fertil. Soc. J..

[77] Zaichick V. (2017). Differences between 66 chemical element contents in normal and cancerous prostate. J. Anal. Oncol..

[78] Foster H., Kennedy G., Maisonneuve P., Krewski D., Ghadiria P. (2008). A case-control study of toenail selenium, mercury, arsenic and cadmium and cancer of the breast, colon and prostate in Montreal. Trends Cancer Res..

[79] Nurchi V.M., Buha Djordjevic A., Crisponi G., Alexander J., Bjørklund G., Aaseth J. (2020). Arsenic toxicity: Molecular targets and therapeutic agents. Biomolecules.

[80] Rambousková J., Krsková A., Slavíková M., Čejchanová M., Černá M. (2014). Blood levels of lead, cadmium, and mercury in the elderly living in institutionalized care in the Czech Republic. Exp. Gerontol..

[81] Cole P., Rodu B. (2005). Epidemiologic studies of chrome and cancer mortality: A series of meta-analyses. Regul. Toxicol. Pharmacol..

[82] Muller C.D., Garcia S.C., Brucker N., Goethel G., Sauer E., Lacerda L.M., Oliveira E., Trombini T.L., Machado A.B., Pressotto A. (2022). Occupational risk assessment of exposure to metals in chrome plating workers. Drug Chem. Toxicol..

[83] Zhang X.-H., Zhang X., Wang X.-C., Jin L.-F., Yang Z.-P., Jiang C.-X., Chen Q., Ren X.-B., Cao J.-Z., Wang Q. (2011). Chronic occupational exposure to hexavalent chromium causes DNA damage in electroplating workers. BMC Public Health.

[84] Krstev S., Knutsson A. (2019). Occupational risk factors for prostate cancer: A meta-analysis. J. Cancer Prev..

[85] Goulart M., Batoréu M.C., Rodrigues A.S., Laires A., Rueff J. (2005). Lipoperoxidation products and thiol antioxidants in chromium exposed workers. Mutagenesis.

[86] Zhang C., Cai K., Feng Q., Xu Y., Zhang Z. (2019). Chromium(VI) promotes cell migration through targeting epithelial-mesenchymal transition in prostate cancer. Toxicol. Lett..

[87] Nsonwu-Anyanwu A., Icha B., Nsonwu M., William M., Emughupogh K., Usoro C. (2022). Assessment of essential and non-essential elements as risk evaluation indices in men with prostate cancer in Calabar South-South Nigeria. Middle East J. Cancer.

[88] Blanc-Lapierre A., Rhazi M., Richard H., Parent M.-E. (2016). O22-3 Occupational Exposure to Chromium, Nickel and Cadmium, and Prostate Cancer Risk and in a Population-Based Case-Control Study in Montreal, Canada. Occup. Environ. Med..

[89] Çelen İ., Müezzinoğlu T., Ataman O.Y., Bakırdere S., Korkmaz M., Neşe N., Şenol F., Lekili M. (2015). Selenium, nickel, and calcium levels in cancerous and non-cancerous prostate tissue samples and their relation with some parameters. Environ. Sci. Pollut. Res..

[90] Chang W.-H., Lee C.-C., Yen Y.-H., Chen H.-L. (2018). Oxidative damage in patients with benign prostatic hyperplasia and prostate cancer co-exposed to phthalates and to trace elements. Environ. Int..

[91] Guntupalli J.N.R., Padala S., Gummuluri A.V.R.M., Muktineni R.K., Byreddy S.R., Sreerama L., Kedarisetti P.C., Angalakuduru D.P., Satti B.R., Venkatathri V. (2007). Trace elemental analysis of normal, benign hypertrophic and cancerous tissues of the prostate gland using the particle-induced x-ray emission technique. Eur. J. Cancer Prev..

[92] Yaman M., Atici D., Bakırdere S., Akdeniz İ. (2005). Comparison of trace metal concentrations in malign and benign human prostate. J. Med. Chem..

[93] Zaichick V., Zaichick S. (2016). Prostatic tissue levels of 43 trace elements in patients with prostate adenocarcinoma. Cancer Clin. Oncol..

[94] Bostwick D.G., Alexander E.E., Singh R., Shan A., Qian J., Santella R.M., Oberley L.W., Yan T., Zhong W., Jiang X. (2000). Antioxidant enzyme expression and reactive oxygen species damage in prostatic intraepithelial neoplasia and cancer. Cancer.

[95] Arsova-Sarafinovska Z., Eken A., Matevska N., Erdem O., Sayal A., Savaser A., Banev S., Petrovski D., Dzikova S., Georgiev V. (2009). Increased oxidative/nitrosative stress and decreased antioxidant enzyme activities in prostate cancer. Clin. Biochem..

[96] Aydin A., Arsova-Sarafinovska Z., Sayal A., Eken A., Erdem O., Erten K., Ozgök Y., Dimovski A. (2006). Oxidative Stress and Antioxidant Status in Non-Metastatic Prostate Cancer and Benign Prostatic Hyperplasia. Clin. Biochem..

[97] Battisti V., Maders L.D.K., Bagatini M.D., Reetz L.G.B., Chiesa J., Battisti I.E., Gonçalves J.F., Duarte M.M.F., Schetinger M.R.C., Morsch V.M. (2011). Oxidative stress and antioxidant status in prostate cancer patients: Relation to gleason score, treatment and bone metastasis. Biomed. Pharmacother..

[98] Oh B., Figtree G., Costa D., Eade T., Hruby G., Lim S., Elfiky A., Martine N., Rosenthal D., Clarke S. (2016). Oxidative Stress in Prostate Cancer Patients: A Systematic Review of Case Control Studies. Prostate Int.

[99] Szewczyk-Golec K., Tyloch J., Czuczejko J. (2015). Antioxidant defense system in prostate adenocarcinoma and benign prostate hyperplasia of elderly patients. Neoplasma.

[100] Tariba Lovaković B., Živković Semren T., Safner T., Gamulin M., Soče M., Pizent A. (2021). Is low-level metal exposure related to testicular cancer?. J. Environ. Sci. Health C Toxicol. Carcinog..

[101] Gào X., Wilsgaard T., Jansen E.H.J.M., Xuan Y., Anusruti A., Brenner H., Schöttker B. (2020). Serum total thiol levels and the risk of lung, colorectal, breast and prostate cancer: A prospective case–cohort study. Int. J. Cancer.

[102] Valko M., Rhodes C.J., Moncol J., Izakovic M., Mazur M. (2006). Free radicals, metals and antioxidants in oxidative stress-induced cancer. Chem. Biol. Interact..

[103] Henkler F., Brinkmann J., Luch A. (2010). The role of oxidative stress in carcinogenesis induced by metals and xenobiotics. Cancers.

[104] Liu J., Qu W., Kadiiska M.B. (2009). Role of oxidative stress in cadmium toxicity and carcinogenesis. Toxicol. Appl. Pharmacol..

[105] Hartwig A. (2010). Mechanisms in cadmium-induced carcinogenicity: Recent insights. Biometals.

[106] Zhu Y., Costa M. (2020). Metals and molecular carcinogenesis. Carcinogenesis.

[107] Ali I., Damdimopoulou P., Stenius U., Halldin K. (2015). Cadmium at nanomolar concentrations activates Raf–MEK–ERK1/2 MAPKs signaling via EGFR in human cancer cell lines. Chem.-Biol. Interact..

[108] Kulkarni P., Dasgupta P., Bhat N.S., Hashimoto Y., Saini S., Shahryari V., Yamamura S., Shiina M., Tanaka Y., Dahiya R. (2020). Role of the PI3K/Akt pathway in cadmium induced malignant transformation of normal prostate epithelial cells. Toxicol. Appl. Pharmacol..

[109] Misra U.K., Gawdi G., Pizzo S.V. (2003). Induction of mitogenic signalling in the 1LN prostate cell line on exposure to submicromolar concentrations of cadmium^+^. Cell. Signal..

[110] Clarkson T.W. (1997). The toxicology of mercury. Crit. Rev. Clin. Lab. Sci..

[111] Fujimura M., Usuki F. (2020). Methylmercury-mediated oxidative stress and activation of the cellular protective system. Antioxidants.

[112] Wang L., Jiang H., Yin Z., Aschner M., Cai J. (2009). Methylmercury toxicity and Nrf2-dependent detoxification in astrocytes. Toxicol. Sci..

[113] Buxton S., Garman E., Heim K.E., Lyons-Darden T., Schlekat C.E., Taylor M.D., Oller A.R. (2019). Concise review of nickel human health toxicology and ecotoxicology. Inorganics.

[114] Zeng X., Jin T., Jiang X., Kong Q., Ye T., Nordberg G.F. (2004). Effects on the prostate of environmental cadmium exposure—A cross-sectional population study in China. Biometals.

[115] van Wijngaarden E., Singer E.A., Palapattu G.S. (2008). Prostate-specific antigen levels in relation to cadmium exposure and zinc intake: Results from the 2001–2002 National Health and Nutrition Examination Survey. Prostate.

[116] Matic B., Rakic U., Dejanovic S., Jovanovic V., Jevtic M., Djonovic N. (2017). Industrially contaminated areas in Serbia as a potential public health threat to the exposed population. Tehnika.

[117] Halamić J., Miko S. (2009). Geochemical atlas of the Republic of Croatia. Croat. Geol. Surv..

